# A dual-modal machine learning framework integrating red blood indices and smartphone-captured microscopic images for β-thalassemia screening

**DOI:** 10.1186/s12911-026-03451-y

**Published:** 2026-03-21

**Authors:** C. R. Wijesinghe, D. C. Liyanaarachchi, P. M. Rathnayake, A. R. Weerasinghe

**Affiliations:** 1 University of Colombo School of Computing, Colombo, Sri Lanka; 2 Department of Pathology, Faculty of Medicine, University of Peradeniya, Peradeniya, Sri Lanka; 3 Informatics Institute of Technology, Colombo, Sri Lanka

**Keywords:** Thalassemia screening, β-thalassemia trait detection, Machine learning in healthcare, Clinical decision support, Red blood cell indices, Blood smear image analysis, Convolutional neural networks (CNN), Transfer learning, Low-resource settings, Smartphone-based microscopy

## Abstract

**Background:**

Thalassemia is the most prevalent single-gene disorder in Sri Lanka, imposing a significant socioeconomic and healthcare burden. Early carrier detection is essential for genetic counselling and the prevention of thalassemia major births. Current screening programs rely on red cell indices, manual examination of blood smears by expert haematologists, and expensive confirmatory tests such as High-Performance Liquid Chromatography (HPLC) or genetic analysis, which limit large-scale applicability in low-resource settings. This study aims to develop a machine learning–based automated screening tool that integrates Red Blood Cell (RBC) indices and blood smear image analysis to support cost-effective and scalable β-thalassemia trait detection.

**Methods:**

In a cross-sectional study of 152 individuals (54 confirmed β-thalassemia trait, 98 negative), 30% of the dataset was allocated for independent testing. The remaining 70% was used for model training, with 5-fold cross-validation for the RBC analysis model and 10-fold cross-validation for the image analysis model. RBC features were classified using a Multi-Layer Perceptron (MLP; scikit-learn), while blood smear images, captured through a conventional microscope using a smartphone camera, were analysed using a transfer learning–based VGG-16 CNN (TensorFlow/Keras). Data balancing and image augmentation (rotation, flipping, brightness variation) were applied to address class imbalance and overfitting. A two-step screening pipeline was proposed, applying RBC analysis first, followed by smear image analysis for RBC-negative cases.

**Results:**

The RBC indices analysis model achieved 88.2% sensitivity (95% CI: 63.6–98.5%) and 92.9% specificity (95% CI: 76.5–99.1%), while the image analysis model reached 88.2% sensitivity (95% CI: 63.6–98.5%) and 64.3% specificity (95% CI: 44.1–81.4%). When integrated sequentially, the combined pipeline achieved 100% sensitivity (95% CI: 80.5–100%) and 60.7% specificity (95% CI: 40.6–78.5%), reducing the overall need for smear preparation by 37.7%.

**Conclusions:**

This enhanced dual-modal screening system provides a highly sensitive, potential to be cost-effective, and practical solution for β-thalassemia carrier detection, enabling mass screening and supporting sustainable prevention strategies in resource-limited settings. With larger, independent, multi-centre validation, it could be integrated into laboratory workflows to expand screening coverage, while ongoing evaluation improves generalisability and inform policy adoption.

**Clinical trial registration:**

Not applicable.

**Supplementary Information:**

The online version contains supplementary material available at 10.1186/s12911-026-03451-y.

## Background

Thalassemia [1] represents a major global health challenge due to its high prevalence and the substantial medical, social and economic burden associated with its management, particularly in low- and middle-income countries (LMICs) such as Sri Lanka [2]. It is a heterogeneous group of genetic disorders characterised by defective synthesis of hemoglobin chains, leading to anemia, ineffective erythropoiesis, and hemolysis [3]. The two principal forms, alpha-thalassemia and beta-thalassemia, vary widely in clinical severity, ranging from asymptomatic microcytosis to life-threatening anemia requiring lifelong transfusion therapy. In Sri Lanka, approximately 3,000 patients [4] depend on regular transfusions, consuming a significant portion of the national healthcare budget [5, 6].

Early screening and diagnosis are critical for preventing thalassemia major births. Thalassemia is inherited as an autosomal recessive disorder. Carriers possess one mutated gene, whereas affected individuals have mutations in both alleles. Consequently, patients are symptomatic within the first two years of life and usually present to hospital with clinical features suggestive of thalassemia. The diagnosis is established based on clinical suspicion and confirmatory investigations, including HPLC demonstrating absent or markedly reduced adult haemoglobin (HbA) with fetal haemoglobin (HbF) as the predominant haemoglobin fraction. Such patients are not detected through population screening programs, as they are present clinically at an early age.

In contrast, thalassaemia carriers are asymptomatic and are commonly identified through carrier detection screening, particularly among relatives of affected patients, as well as through school-based screening programs and premarital screening initiatives.

Current screening methods in Sri Lanka involve assessing red blood cell indices from a full blood count (FBC), peripheral blood smear examination by haematologists, and confirmatory tests such as High-Performance Liquid Chromatography (HPLC). While HPLC and genetic analysis provide very high diagnostic accuracy, these methods are time-consuming, expensive, and dependent on specialised expertise, making widespread implementation challenging. Despite Sri Lanka’s existing prevention program, the birth rate of thalassemia major cases remains inadequately controlled, demonstrating the need for more effective screening strategies. A simple, cost-effective, yet highly sensitive screening approach is therefore needed—one that can efficiently identify individuals at risk and prioritise them for confirmatory testing without overburdening clinicians and laboratory personnel. Such a method should ideally function with basic RBC data and blood smear images, even in rural primary care settings lacking haematologists.

Machine learning has emerged as a powerful tool in medical diagnostics, offering potential improvements in efficiency, accuracy, and cost reduction. Various machine learning approaches have been applied to haematological disorders, but research on thalassemia screening remains limited. Previous studies have either focused on RBC-based classification models or blood image analysis, but not both simultaneously. Integrating these two approaches could significantly enhance diagnostic reliability by combining morphological and haematological features.

However, a critical gap persists: current digital tools do not provide an integrated ML pipeline capable of processing both numerical RBC indices and smear-image features, nor are they designed for practical deployment in LMICs. Many existing systems require high-quality laboratory imaging, powerful computing resources, or specialised environments, making them unsuitable for decentralised or primary-care screening.

In LMICs, the broader deployment of AI tools is challenged by variability in smartphone imaging quality, limited computational capacity, intermittent internet connectivity, and difficulties integrating digital tools into routine health workflows. Addressing these context-specific barriers is essential for achieving real-world impact. This study presents an enhanced screening framework explicitly designed to operate on average-priced smartphones, without requiring expensive high-tech equipment or internet connectivity. The system features a simple user interface, enabling healthcare workers to quickly and accurately screen individuals, triage a small number for confirmatory testing, and facilitate decentralised testing with minimal infrastructure. Embedding such a tool within national screening pathways has significant potential to enhance prevention efforts, particularly as Sri Lanka prepares to implement premarital thalassemia screening policies.

This study aims to develop a machine-learning–based screening system optimised for high sensitivity and practical implementation, leveraging convolutional neural networks, transfer learning, and complementary ML techniques. A notable innovation of the present study is the development of an integrated pipeline that fuses Red Blood Cell (RBC) indices with CNN-derived blood smear image features into a unified, deployable screening system. The pipeline is explicitly designed to operate on average-priced smartphones, without requiring expensive high-tech equipment or internet connectivity, and features a simple user interface that enables healthcare workers to quickly and accurately screen individuals, triage a small number for confirmatory testing, and facilitate decentralised testing with minimal infrastructure, enabling use even in rural primary care settings without haematologists.

This approach is relevant not only for thalassemia prevention but also for broader digital health implementation, as it demonstrates how AI tools can be adapted for low-resource settings, embedded into existing care pathways, and scaled to support national public-health strategies. By offering a sensitive, efficient, and sustainable solution, the proposed system has significant potential to strengthen thalassemia screening, particularly as Sri Lanka prepares to implement premarital screening policies and expand population-level preventive interventions.

Machine learning techniques have increasingly been applied to automate microscopic image analysis in haematological cytology, addressing critical challenges in blood image processing. The accuracy of these methods depends on multiple factors, including slide preparation, staining quality, and digitisation settings. Optimising algorithm performance often requires standardised image acquisition protocols to ensure consistency and reliability across datasets [7]. One major challenge in blood smear analysis is detecting rare cells in peripheral blood. While analysing common blood cells can be done with small sample sections, the same approach may not be effective for rare cell detection due to limited imaging, potentially leading to false negatives. To mitigate this, studies emphasise the need for analysing larger slide areas, where image acquisition speed becomes a crucial factor [8].

To transition from research to practical application, systematic validation strategies must be established in laboratory environments. Defining comprehensive screening protocols will be essential to maximise the potential of automated recognition methodologies, making them suitable for clinical deployment [9].

Several studies have focused on automated thalassemia detection. Sandanayake et al. [10] developed a system to identify thalassemia using RBC images and machine learning. The system extracted features such as cell shape, size, and colour using edge detection (Canny), Hough Transform, and ellipse fitting, alongside quantitative parameters including RBC count, mean corpuscular volume (MCV), mean corpuscular haemoglobin (MCH), mean corpuscular haemoglobin concentration (MCHC), red blood cell distribution width (RDW), and haemoglobin content. Classification was performed using Artificial Neural Networks (ANN) and Support Vector Machines (SVM), achieving high accuracy (89.4% for females and 97% for males). The approach included a web interface for patient interaction, improving accessibility. However, the system relied solely on RBC morphological features without integrating RBC indices or other haematological parameters, limiting potential diagnostic accuracy. Validation was performed on a relatively small and gender-specific dataset, and modern deep learning methods such as CNNs or transfer learning were not explored.

Khan et al. [11] developed a deep learning approach using Hb electrophoresis images, achieving up to 95.8% accuracy with pre-trained CNNs and confirming the model’s focus on relevant haemoglobin bands. Although HPLC remains faster, more accurate, and convenient for confirmation, this study provides insights into transfer learning, pre-processing techniques, and interpretability methods such as Score-CAM, which are valuable for developing image-based screening models like ours.

ThalPred is a machine learning tool designed to discriminate thalassemia trait (TT) from iron deficiency anaemia (IDA) using routine RBC parameters. Differentiating these two causes of hypochromic microcytic anaemia is challenging, especially in Thailand, due to the variability of RBC indices across populations. Using 186 adult samples, multiple models, including decision trees, random forests, SVM, and neural networks, were tested. While traditional indices like KF2 and RI performed moderately well, machine learning models, particularly SVM, achieved higher accuracy and robustness even on independent validation sets. Additionally, interpretable rules extracted from random forests enabled clinicians to screen using simple thresholds of Hb, RBC, RDW, and MCHC. Compared to our model, ThalPred emphasises the value of SVM for imbalanced datasets and highlights the importance of interpretability in clinical tools, reinforcing the benefit of combining robust algorithms with transparent decision rules.

Lin et al. [12] used a Mask R-CNN-based deep learning model to detect and segment thalassemic RBCs from quantitative phase images, achieving 97.8% classification accuracy. Their approach combined instance segmentation with feature-based classifiers to extract single-cell morphology and haemoglobin content, providing detailed interpretability. However, the reliance on specialised digital holographic microscopy limits accessibility and real-time applicability. In contrast, our model uses full blood count parameters and blood smear images captured through a mid-range smartphone via a microscope eyepiece, offering greater accessibility and feasibility for widespread use. Insights from Lin et al. underscore the value of combining morphological information with quantitative features, guiding our approach to integrate image- and parameter-based data for accurate and interpretable thalassemia screening.

Sadiq et al. [13] proposed an ensemble model, SGR-VC, combining SVM, Gradient Boosting Machine (GBM), and Random Forest (RF) to classify carriers and non-carriers using a dataset of 5,066 patients from the Punjab Thalassemia Prevention Program. The model achieved 93% accuracy, precision, recall, and F1-score, outperforming individual classifiers. While earlier works primarily focused on distinguishing β-thalassemia from iron deficiency anaemia or on specific subgroups such as women of fertile age, SGR-VC demonstrates a more generalisable approach across genders and age groups. However, gaps remain in exploring deep learning methods, regression-based models, and datasets from diverse geographic populations, which could further improve predictive power. Insights from this study highlight the effectiveness of ensemble approaches in leveraging complementary strengths of tree-based and probability-based classifiers, and the importance of balanced, normalised datasets for robust carrier detection. Compared to our model, SGR-VC serves as a benchmark showing that combining multiple classifiers can reduce misclassification and improve precision, providing a cost-effective, rapid screening tool suitable for large-scale public health applications.

Purwar et al. [14] proposed a novel approach for thalassemia detection by fusing deep image features from blood smear images with clinical features from RBC indices. Using AlexNet CNN to extract morphological features and ten clinical parameters, such as RBC count, haemoglobin, MCV, and haemoglobin A2, the study applied PCA to reduce redundancy and prevent overfitting in the high-dimensional fused feature set (1011 features) relative to a small sample size (20 patients). Classification using Naive Bayes, Random Forest, and K-NN achieved 99 ± 1% accuracy with 100% sensitivity and specificity. While demonstrating the power of multimodal feature fusion, gaps remain in scaling to larger, more diverse populations and in exploring end-to-end deep learning models without feature engineering. Compared to our model, this study highlights the performance gains achievable when combining clinical and morphological information. Insights suggest that integrating heterogeneous data sources with dimensionality reduction can substantially improve detection accuracy even with limited datasets, providing a practical framework for automated thalassemia screening.

Collectively, these studies demonstrate the promise of machine learning and deep learning for automated thalassemia detection. While prior work often focuses either on blood smear image analysis or RBC-based classification, few approaches integrate both modalities. Combining morphological and haematological features enhances diagnostic reliability and supports scalable, patient-centred screening systems, particularly in resource-limited settings such as Sri Lanka.

## Methods

### Study design and sample collection

Following ethical clearance, data were collected from the individuals attending the National Thalassemia Centre in Kurunegala, Sri Lanka. Participants were recruited through three main pathways: voluntary screening, which included individuals who independently sought thalassemia carrier testing; referrals from primary care physicians or hospital wards for those with clinically suspected anaemia or microcytic hypochromic red cell indices; and mass screening programs conducted in schools across the Kurunegala district, particularly targeting adolescent populations. The routine diagnostic workflow at the centre was used for the study without any alterations.

In routine (non-programmatic) screening, a structured clinical history was first obtained, followed by a Full Blood Count (FBC). Individuals with microcytic, hypochromic red cell indices (Mean Corpuscular Volume [MCV] < 77 fL and Mean Corpuscular Haemoglobin [MCH] < 27 pg) were further investigated by peripheral blood smear examination. Smears were prepared by trained laboratory technicians from venous blood collected in Ethylenediaminetetraacetic acid (EDTA) tubes, stained using the Leishman, and examined by haematologists for morphological features suggestive of thalassemia, including target cells, pencil cells, and tear-drop cells. Suspected cases were subjected to quantitative haemoglobin analysis using High-Performance Liquid Chromatography (HPLC), which is the standard confirmatory test for β-thalassemia in Sri Lanka. Individuals with an HbA₂ level > 3.5% were classified as β-thalassemia carriers.

Following completion of diagnostic confirmation, Full Blood Count (FBC) reports, blood smears, and HPLC results, materials that would otherwise be discarded, were collected for analysis. Sensitive personal identifiers were removed, and only anonymised report numbers were retained to link FBC, smear, and HPLC data.

Written informed consent was obtained from all participants prior to data use. For minors and individuals unable to provide consent, proxy consent was obtained from parents or legal guardians. All data were stored securely accessible only to authorised research personnel, and documentation of consent and ethical approval will be made available to the ethics review committee or journal upon request.

Exclusion criteria included incomplete FBC reports, inability to retrieve the corresponding HPLC result, or absence of usable blood smear images. In addition, individuals who were HPLC-negative for β-thalassaemia but subsequently underwent further investigations for other haemoglobinopathies or alternative causes of anaemia were excluded to avoid diagnostic heterogeneity. No exclusions were made on the basis of prior blood transfusions or iron therapy.

### Population characteristics and generalisability considerations

Although screening services are available in multiple tertiary hospitals and dedicated haematology clinics across the country, the National Thalassemia Centre at Teaching Hospital Kurunegala functions as the largest national referral and screening hub. It hosts the highest number of registered patients and conducts the most extensive screening programmes in Sri Lanka, reflecting the highest reported regional prevalence of thalassaemia in areas surrounding Kurunegala. As a result, the study cohort is likely representative of the population most affected by β-thalassaemia within the national context.

Age groups were approximately equally represented, with a near-balanced sex distribution (79 females, 73 males), reducing the likelihood of sex- or age-related sampling bias within the cohort. However, the dataset was collected from a single geographical region, and demographic characteristics beyond age and sex, such as ethnicity, socioeconomic background, and place of residence, were not recorded. These factors may or may not influence red blood cell indices or peripheral smear morphology, and to date, no scientific evidence has demonstrated systematic variation in these haematological parameters attributable to such demographic variables in Sri Lankan populations. Nonetheless, the absence of these data limits the ability to fully assess potential demographic bias.

Furthermore, while some sources of variability were incorporated into the dataset through differences in operators and acquisition devices, the model has not yet been evaluated in populations served by other screening centres, laboratories using different equipment, or regions with differing genetic backgrounds. Therefore, future work should aim to include a more demographically diverse cohort through a multi-centre, nationwide study to enhance external validity and support broader clinical deployment.

### Data acquisition

Two types of data were acquired: Red Blood Cell Indices and Blood Smear Imaging.

### Red blood cell indices

The following red cell indices and haematological parameters that represent the core haematological abnormalities characteristic of β-thalassemia were collected for each participant: Haemoglobin (Hb), Red Blood Cell count (RBC), Mean Corpuscular Volume (MCV), Mean Corpuscular Haemoglobin (MCH), Mean Corpuscular Haemoglobin Concentration (MCHC), and Red cell Distribution Width (RDW). Age and sex were added to account for potential demographic effects.

The study cohort comprised 152 individuals recruited through voluntary screening, clinical referrals, and school-based mass screening programs. Ages ranged from 1 to 63 years (mean ± SD: 22.6 ± 12.1 years). Among β-thalassemia trait (BTT)–positive individuals (*n* = 54), there were 27 males and 27 females, while the BTT-negative group (*n* = 98) included 46 males and 52 females. Ethnicity was not explicitly recorded; however, participants were recruited from the Kurunegala district, which largely reflects the ethnic composition of the broader Sri Lankan population. Prior population-based studies in Sri Lanka have reported minimal variation in β-thalassemia trait prevalence across major ethnic groups (2.1% Sinhalese, 1.5% Tamils, 1.0% Moors) (Premawardhena & Madushanka, 2022), suggesting that the absence of explicit ethnic labels is unlikely to introduce introduce significant population stratification or systematic bias in this context.

β-thalassemia is an autosomal recessive condition, so carrier status is not influenced by sex, which is reflected in the balanced male–female distribution in our dataset and minimizes sex-related bias. Regarding age, β-thalassemia major presents clinically in infancy and is not part of population screening; these cases were excluded. Carriers, however, are asymptomatic and typically identified through school, premarital, or family screening, which explains the wide age range in our dataset. Therefore, the age and sex distribution observed is appropriate for carrier screening and does not introduce demographic bias.

Statistical tests confirmed that age and sex were comparable between BTT-positive and BTT-negative groups (age: *p* = 0.87; sex: *p* = 0.737), indicating no demographic imbalance. To further minimise bias, age and sex were included as features in the haematological model, and all cross-validation was performed at the patient level to prevent information leakage.

Table 1 shows a descriptive summary of the study dataset. For each RBC parameter and age, the mean ± standard deviation (SD) and observed range are presented for the total cohort.

**Table 1:** Descriptive summary of the study dataset: Red Blood Cell indices and age stratified by β-thalassemia trait (BTT) status and sex

Parameter	Total Dataset	Positive (BTT)			Negative (Normal)		
		Male	Female	Total	Male	Female	Total
MCV	71.46 ± 10.63 (51.4–90.5)	59.57 ± 4.39 (51.4–70.2)	60.21 ± 4.06 (53.9–74.4)	59.89 ± 4.20 (51.4–74.4)	77.26 ± 8.14 (54.3–89.9)	77.71 ± 6.96 (56.1–90.5)	77.51 ± 7.47 (54.3–90.5)
MCH	23.47 ± 4.22 (15.1–33.0)	19.01 ± 1.42 (16.4–21.6)	19.09 ± 1.34 (17.0–23.9)	19.05 ± 1.37 (16.4–23.9)	25.75 ± 3.56 (15.1–31.2)	25.80 ± 3.01 (17.7–33.0)	25.78 ± 3.25 (15.1–33.0)
MCHC	32.59 ± 1.94 (14.3–35.7)	31.97 ± 0.64 (30.5–32.9)	31.83 ± 0.76 (29.6–33.2)	31.90 ± 0.70 (29.6–33.2)	33.25 ± 1.58 (27.9–35.7)	32.72 ± 2.67 (14.3–34.9)	32.96 ± 2.26 (14.3–35.7)
Hb	12.00 ± 2.11 (5.7–19.9)	11.24 ± 1.63 (8.5–13.8)	10.58 ± 1.07 (8.5–12.4)	10.91 ± 1.40 (8.5–13.8)	13.30 ± 2.59 (5.7–19.9)	12.00 ± 1.63 (9.4–17.3)	12.57 ± 2.19 (5.7–19.9)
RBC	5.16 ± 0.73 (3.2–7.1)	5.91 ± 0.77 (3.9–7.1)	5.52 ± 0.46 (4.6–6.1)	5.72 ± 0.65 (3.9–7.1)	5.12 ± 0.60 (3.2–6.3)	4.69 ± 0.52 (3.5–5.8)	4.88 ± 0.60 (3.2–6.3)
RDW	15.56 ± 2.17 (11.4–24.7)	17.31 ± 1.24 (14.8–20.0)	16.45 ± 1.23 (12.5–19.0)	16.88 ± 1.30 (12.5–20.0)	14.39 ± 1.74 (11.4–21.0)	15.24 ± 2.48 (12.7–24.7)	14.86 ± 2.21 (11.4–24.7)
Age	22.29 ± 12.85 (1.0–63.0)	24.02 ± 15.81 (1.0–63.0)	19.29 ± 12.46 (2.8–45.0)	21.65 ± 14.30 (1.0–63.0)	22.37 ± 13.21 (1.0–48.0)	22.84 ± 11.22 (1.0–59.0)	22.63 ± 12.07 (1.0–59.0)

Key observations from the dataset indicate that BTT-positive individuals exhibit markedly lower MCV values and MCH values compared to BTT-negative participants, who show higher averages for both parameters. Additionally, BTT-positive individuals demonstrate a higher RBC count than the negative group reflecting the compensatory erythropoiesis characteristic of thalassemia carriers. RDW and MCHC values showed additional distinctions between groups. BTT-positive individuals had higher RDW values compared to BTT-negative participants, indicating greater variability in red cell size. Differences in MCHC were more modest, although slightly lower values were observed among BTT-positive cases compared with the negative group.

The age distribution of participants ranged from 1 to 63 years, with similar mean ages across groups. BTT-positive individuals had a mean age of 21.65 ± 14.30 years, closely aligned with the mean age of 22.63 ± 12.07 years observed in the negative group, suggesting no notable age-related sampling bias. Sex distribution was also balanced across the cohort, with male and female participants represented in approximately equal proportions in both BTT-positive and BTT-negative groups. Minor sex-related differences were noted Hb and RBC, with males showing slightly higher values. However, key screening parameters such as MCV and MCH demonstrated minimal variation by sex, indicating that these indices are largely independent of sex within this dataset.

The dataset provides a comprehensive representation of RBC indices for both BTT-positive and BTT-negative individuals, making it suitable for training and validating the dual-modal machine learning pipeline. The distribution of values also highlights the discriminative potential of CBC indices for initial β-thalassemia screening.

### Blood smear imaging

Blood smear images were captured using two approaches - Smartphone-assisted microscopy and Standard microscope camera.

**Smartphone-assisted microscopy** – Three different smartphones were used: Samsung Galaxy M21, Apple iPhone 15, and Xiaomi Redmi Note 13. Three operators independently captured images, with slides randomly assigned in approximately equal proportions to simulate real-world variability in community and laboratory settings. Each phone camera was manually aligned with the microscope eyepiece without customised adapters or camera settings, and the smears were focused under the ×100 oil immersion objective. The microscope lamp served as the sole illumination source, and no exposure, colour, or white-balance calibration was performed prior to acquisition. Images were saved in the native formats of the devices (HEIC or PNG) and subsequently converted to JPEG during preprocessing. This method emulates routine haematological practice, with the smartphone effectively substituting for the human eye.

**Standard microscope camera** – An AxioCam ERc5s camera attached to a standard light microscope was used to acquire reference-quality images at the same ×100 magnification. Relevant camera metadata, including pixel accuracy (0.0039 mm), image size (up to 5712 × 4284 pixels), light path, and lens information, were retained for reproducibility.

**Fig. 1:**
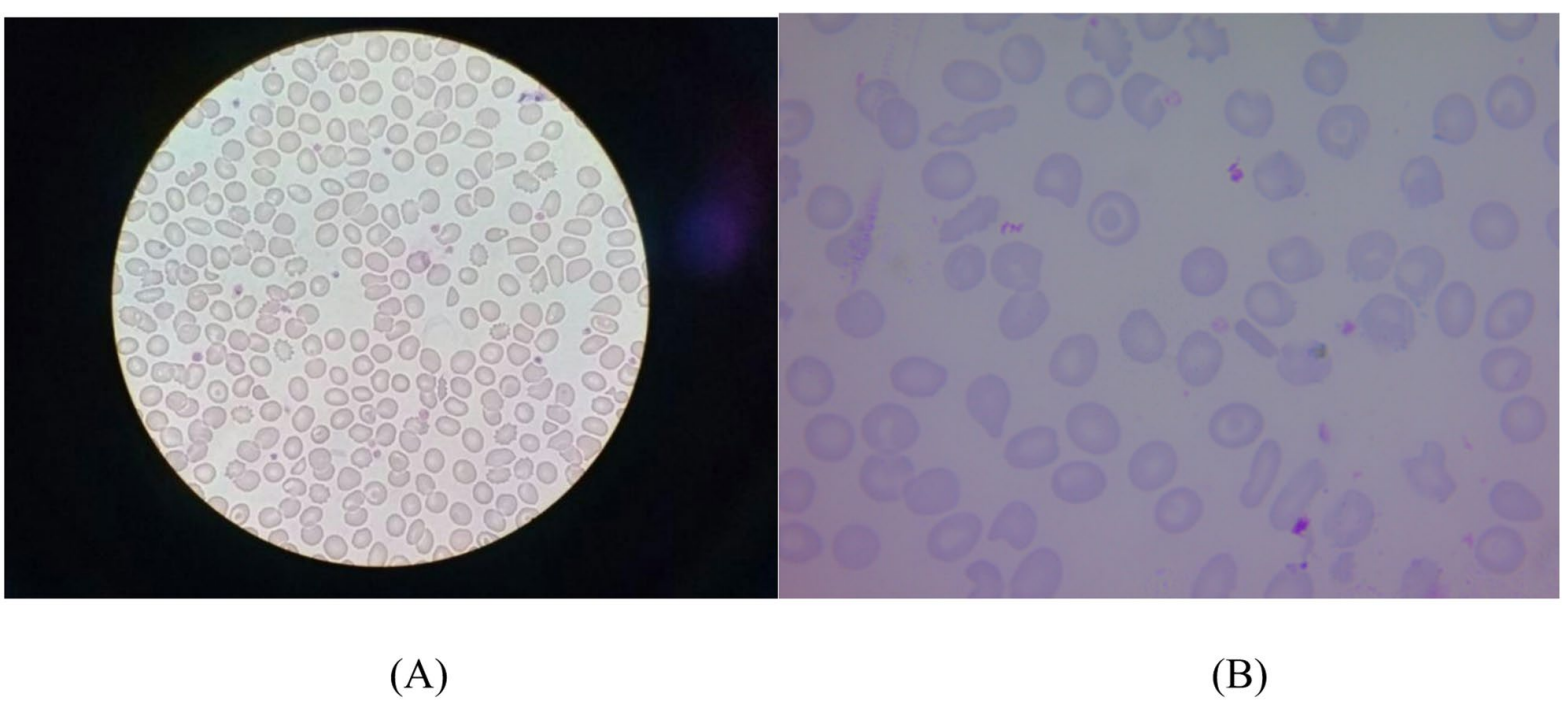
Representative blood smear images used in the study. (**A**) Smartphone microscopy; (**B**) Standard microscope camera

For each smear, 25–30 non-overlapping images were obtained from the morphological “zone of interest,” i.e., the area between the body and tail of the smear where red cells are evenly distributed, non-overlapping, and morphologically intact. The head and tail regions, where cells are either too crowded or distorted, were excluded following standard haematology practice. Smear examination was performed systematically using a Z-pattern scanning approach to ensure comprehensive coverage. Representative blood smear images used in the study is shown in Fig. 1.

Captured images were reviewed, and any blurred or artefact-containing images were excluded. The final dataset comprised 5,198 high-quality images from 152 blood smear samples, each sample labelled as thalassemia carrier or non-carrier according to the corresponding HPLC results.

### Data preprocessing

#### Haematological data (Red blood cell indices)

The FBC reports obtained from the laboratory information system were exported into a structured format and underwent several preprocessing steps. Personal identifiers were removed to ensure anonymisation, with only the report ID retained to match smear images and HPLC results. FBC reports containing incomplete or missing red cell indices were excluded, and any outliers falling outside physiologically plausible limits were flagged and manually verified against the original records. Numerical variables, including MCV, MCH, MCHC, haemoglobin (Hb), RDW, and RBC count, were normalised using z-score standardization. Patient sex was encoded as a binary variable (0 = female, 1 = male). HPLC-confirmed carrier status served as the ground truth label, with carriers coded as 1 and non-carriers as 0. These steps resulted in a structured tabular dataset of RBC indices aligned with each participant’s corresponding smear images.

### Blood smear images

**Fig. 2:**
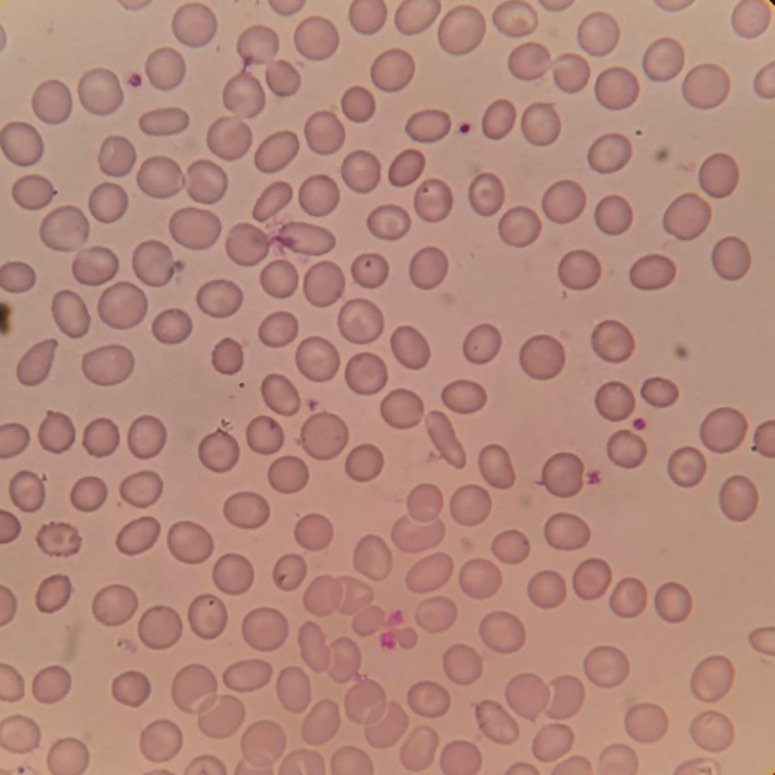
Blood smear image after pre-processing

The raw smear images underwent several preprocessing steps to ensure consistency and suitability for deep learning analysis. Initial quality control filtering was performed to remove images affected by motion blur, poor focus, or uneven staining, using both visual inspection and automated quality checks. To minimise background artefacts, a rectangular Region of Interest (ROI) corresponding to the largest inscribed area within the circular microscope field was extracted using the Hough Circle Transform in OpenCV, allowing automatic cropping of the maximal area containing red blood cells. All images were then resized to 224 × 224 pixels to meet the input requirements of the convolutional neural network (CNN) and maintain computational efficiency. To address class imbalance and improve model generalisation, data augmentation techniques—including random rotations (± 15°), horizontal and vertical flips, minor translations and zooming, and controlled brightness and contrast variations—were applied (Fig. 2). Following preprocessing, the dataset was divided into training (70%) and independent testing (30%) subsets, with patient-level stratification to prevent data leakage by ensuring that images from the same individual did not appear across different partitions.

### Model architecture and training

The proposed model architecture was designed with careful consideration of the requirements of a population-level screening tool. In such settings, maximising sensitivity is paramount to ensure that no β-thalassemia carrier is missed, even at the expense of specificity. A false-negative result could delay diagnosis and increase the risk of undetected carrier marriages, whereas false positives can be resolved at the confirmatory testing stage.

In addition to achieving high diagnostic accuracy, the architecture was intentionally designed to meet the practical challenges of real-world healthcare delivery, especially in resource-limited and rural settings where trained haematologists and advanced laboratory infrastructure may be scarce. The system therefore aimed to minimise the workload on clinicians and laboratory staff by reducing the need for unnecessary smear preparation and microscopic examination, while also lowering overall costs and reagent consumption to support large-scale screening initiatives. Furthermore, portability and scalability were prioritised to enable deployment across multiple platforms—from mobile devices to desktop systems—ensuring broad accessibility. Finally, the pipeline was optimised for rapid turnaround, delivering near real-time results suitable for screening during outreach activities and school-based screening programmes.

### Overview of the dual-modal framework

A two-step dual-modal framework was implemented specifically for population-level screening, not as a diagnostic replacement. It has two architecturally distinct components: an MLP model that processes numerical RBC indices and a CNN that analyses raw smear images. These modalities differ in input format, feature representation, and model architecture, but are integrated sequentially to improve screening performance.

**Step 1** of the screening pipeline involves haematological analysis based RBC indices together with patient age and sex. This stage was optimised for high specificity to ensure that individuals flagged as positive could be confidently referred directly for confirmatory HPLC testing without requiring additional intermediate assessments. Only those classified as negative at this stage proceed to the secondary evaluation.

**Step 2** of the pipeline focuses on morphological screening using blood smear images. For individuals not identified as positive in the first stage, peripheral blood smears are prepared, digitised, and analysed using a CNN-based model trained to detect subtle morphological features associated with thalassemia. This stage was intentionally designed to maximise sensitivity, ensuring that borderline or atypical cases are captured and the risk of missed carriers is minimised.

By integrating the two modalities in a sequential architecture, the pipeline ensures high diagnostic reliability while maintaining operational efficiency. At the same time, the overall workload for laboratory staff is significantly reduced, as only a small subset of individuals who screen negative initially require smear preparation and further microscopic analysis. This design also optimises time, cost, and reagent consumption, making the workflow well suited for mass-screening programmes where preserving resources without compromising clinical robustness is essential.

**Fig. 3:**
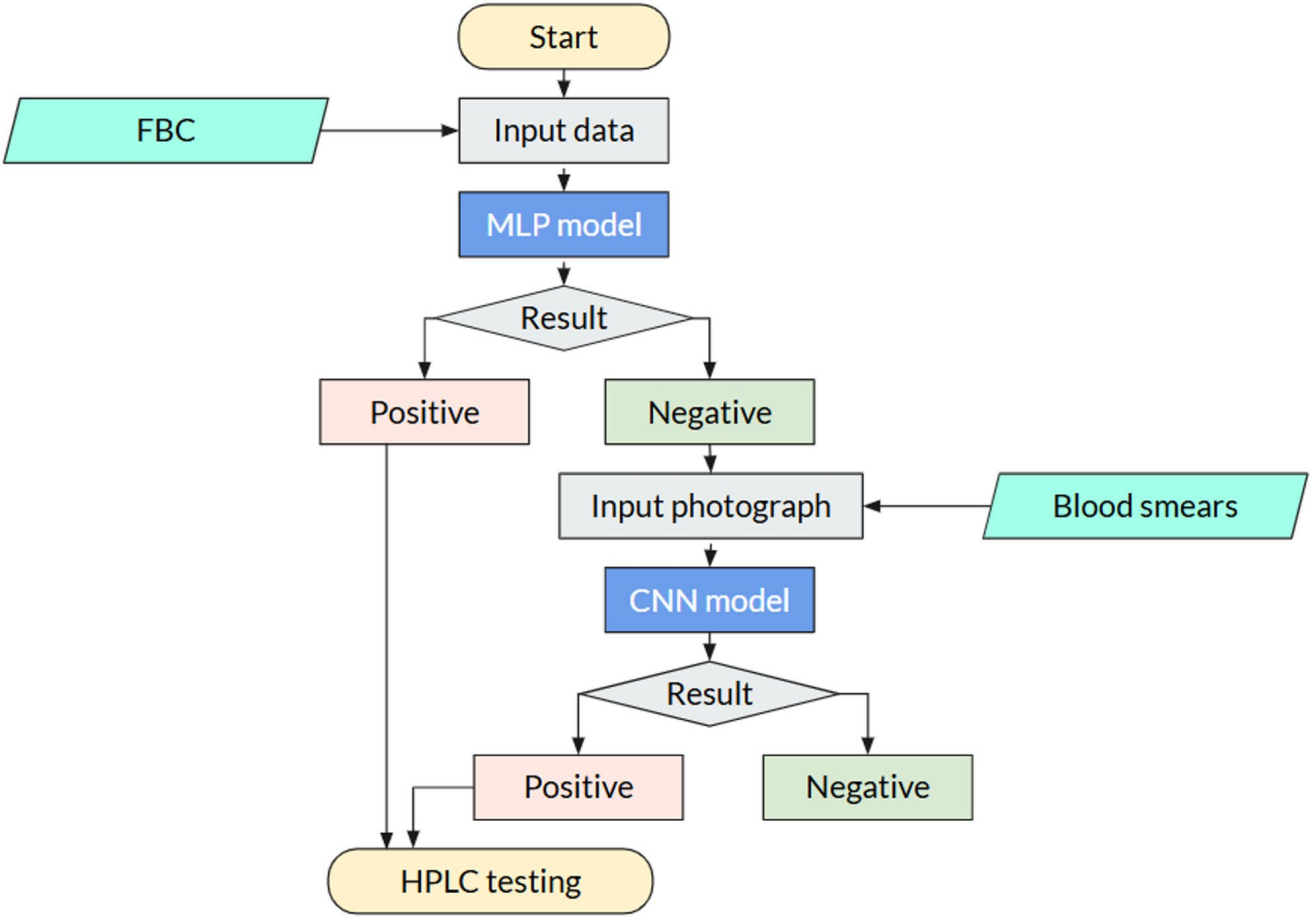
Schematic overview of the dual-modal machine learning pipeline for β-thalassemia screening: MLP model and CNN Model

The dual-modal strategy is shown in Fig. 3.

### Processing of red blood cell indices and blood smear images

Septs followed in processing the Blood Cell Indices and Blood Smear Images are discussed below.

### Processing red blood cell indices

The first component of the dual-modal model processes Red Blood Cell (RBC) indices, along with patient age and sex.

The preprocessing and model selection workflow included the following steps:

I. Dimensionality Reduction (PCA):
  Principal Component Analysis (PCA) was applied to the RBC dataset to identify the most informative combinations of red cell indices. PCA helped reduce redundancy between correlated parameters while retaining the variance most relevant for discriminating carriers from non-carriers. The resulting principal components were analysed to guide feature selection for downstream model training.

II. Algorithm Evaluation:
  To identify the optimal classifier for RBC-based screening, nine machine learning algorithm**s** were trained and compared using different permutations of RBC parameters: Logistic Regression (LR), k-Nearest Neighbors (k-NN), Decision Tree (DT), Random Forest (RF), Gradient Boosting Machine (GBM), AdaBoost, Support Vector Machine (SVM), Naive Bayes (NB), Multilayer Perceptron (MLP). Each model was evaluated on training and validation sets using cross-validation to ensure robustness, and performance metrics, including accuracy, precision, recall, and area under the ROC curve (AUC), were compared.

III. Feature Selection & Optimisation:
  Based on performance, the most informative subset of RBC parameters was selected to maximise specificity in this first step, ensuring that predicted positives could be confidently sent for confirmatory HPLC without unnecessary smear preparation. Hyperparameters for each algorithm were tuned using grid search or Bayesian optimisation to achieve optimal predictive performance.

### Processing blood smear images

For model development, blood smears were prepared for 152 out of 163 participants, comprising 98 negative and 54 positive cases. All images underwent the preprocessing steps described previously, including ROI extraction using Hough Circle Transform, resolution standardisation (224 × 224 pixels), and data augmentation to increase variability and reduce class imbalance.

The image component employed transfer learning to leverage pretrained convolutional neural networks (CNNs) for feature extraction. Multiple architectures were evaluated: VGG16, ResNet50, DenseNet121, MobileNetV2 and ConvNeXtTiny. Each architecture was fine-tuned and hyperparameters were systematically varied using grid search or random search to identify the optimal configuration for each model.

Models were trained using training and validation subsets, with patient-level stratification to prevent data leakage. Performance metrics, including accuracy, sensitivity, specificity, F1-score, and area under the ROC curve (AUC), were calculated to identify the model with the best sensitivity, ensuring that borderline and atypical positive cases were reliably detected.

The selected image model forms the second-stage screening component.

### Model training and optimisation

Training and optimization of each model is described below.

### RBC indices model

The processed RBC dataset was used to train a machine learning model for β-thalassemia carrier classification. The model was implemented in Python 3.9, using scikit-learn v1.2, along with NumPy, Pandas, Matplotlib, and Seaborn for evaluation and visualisation. The MLP architecture comprised hidden layers with ReLU activation, fine-tuned to optimise performance for binary classification. To ensure balanced representation of carriers and non-carriers and to prevent data leakage, stratified 5-fold cross-validation was employed throughout the optimisation process. Performance of the model was evaluated using a comprehensive set of custom metrics, including sensitivity, specificity, accuracy, F1-score, ROC–AUC, and the Matthews correlation coefficient.

### Blood smear image model

The second component of the dual-modal model processes blood smear images and was implemented in Python 3.9 using TensorFlow/Keras 2.x alongside standard scientific libraries such as NumPy, Pandas, and Matplotlib. This component was designed to ensure patient-level stratified training and evaluation, preventing data leakage across folds or between images belonging to the same individual. Pretrained convolutional neural networks were used as feature extractors. Their base layers were initially frozen, and a custom classifier head was added, consisting of a global average pooling layer, a dense layer with 256 ReLU-activated units, a dropout layer with a rate of 0.5, and a final softmax output layer with two units for binary classification. After initial training, the top four layers of each CNN backbone were unfrozen for fine-tuning. Training followed a two-stage strategy: 50 epochs with a frozen backbone using the Adam optimiser (learning rate = 0.001), followed by 50 fine-tuning epochs with a reduced learning rate of 1e-5. Images were processed at 224 × 224 resolution with a batch size of 32, and extensive data augmentation—such as random flips, brightness and contrast adjustments, saturation shifts, and rotations—was applied to improve generalisation.

To maintain rigorous reproducibility, patient-level splitting was enforced using train_test_split to ensure that no images from the same patient appeared in both training and validation sets. A custom PatientFolderDataGenerator, implemented as a subclass of tf.keras.utils.Sequence, handled batch generation, preprocessing, augmentation, and GPU-accelerated loading. Training incorporated callbacks including EarlyStopping (patience = 10, monitoring validation accuracy), ReduceLROnPlateau (factor = 0.5, patience = 5), and ModelCheckpoint for saving the best-performing model. No tuning decisions were informed by the independent test dataset, thereby preserving its validity as an unbiased external.

### Probabilistic calibration of MLP and CNN models

To evaluate the reliability of predicted probabilities, we performed calibration analysis on both the MLP (tabular) and CNN (image-based) models. Two key metrics were used: Brier Score, measuring the accuracy of probabilistic predictions and Expected Calibration Error (ECE).

### Implementation and deployment of dual-modal pipeline

The proposed dual-modal pipeline was designed with practical deployment in low-resource and primary care settings in mind. A key feature of the system is that it does not require specialised imaging hardware; smartphone-based microscopy was performed using standard laboratory microscopes without custom adapters, modified optics, or controlled imaging environments. This approach reflects the conditions under which peripheral smear evaluation is routinely carried out in many regional hospitals and rural clinics in Sri Lanka, enabling potential adoption without additional capital cost.

In a real-world workflow, peripheral smear images captured using a smartphone or existing microscope camera could be uploaded to a local workstation or mobile application interface, where automated image analysis and FBC feature processing would be performed. The computational requirements of the final model allow inference to be executed either on-device for newer smartphones or via a low-resource edge or cloud-based server, making the system adaptable to settings with variable technical infrastructure. This supports potential deployment in rural primary care clinics that lack on-site haematologists, providing decision support for early carrier screening and referral.

The intended operational use-case involves assisting healthcare workers by flagging individuals with a high likelihood of β-thalassaemia trait, thereby prioritising confirmatory testing and improving screening throughput. However, the current implementation remains a research prototype, and several constraints must be addressed prior to clinical integration. These include regulatory approval, standardisation of smear preparation and staining quality, operator training for consistent image capture, and integration with electronic laboratory information systems. Connectivity limitations in remote settings may also affect workflows reliant on cloud-based processing.

Future development will focus on a full mobile application interface, streamlined clinician-facing reporting, and incorporation into existing national thalassaemia screening programmes. Prospective field evaluation and usability testing will be required to ensure that the system is safe, scalable, and operationally feasible in real-world clinical environments.

### Primary endpoint

The proposed framework was developed as a screening and triage tool for β-thalassemia trait detection prior to confirmatory high-performance liquid chromatography (HPLC) testing. Accordingly, model evaluation prioritised metrics relevant to population screening rather than overall classification accuracy. The primary endpoint of the study was sensitivity, defined as the ability of the model to correctly identify β-thalassemia carriers. As a co-primary endpoint, negative predictive value (NPV) was assessed to ensure reliable exclusion of non-carriers during screening. These metrics were selected to minimise missed carriers while maintaining safe clinical triage. All classification results were computed using a predefined probability threshold of 0.5, where predicted probabilities ≥ 0.5 were classified as carrier positive. Performance metrics reported across all models and pipeline evaluations are aligned with this predefined screening objective.

## Results

### RBC-only model (MLP)

The PCA results revealed that MCV and MCH contributed most strongly to the first two principal components, indicating that these parameters alone capture the majority of the variance distinguishing β-thalassemia trait from normal individuals. This observation aligns with clinical knowledge, as microcytosis (low MCV) and hypochromia (low MCH) are hallmark features of thalassemia carriers.

**Fig. 4:**
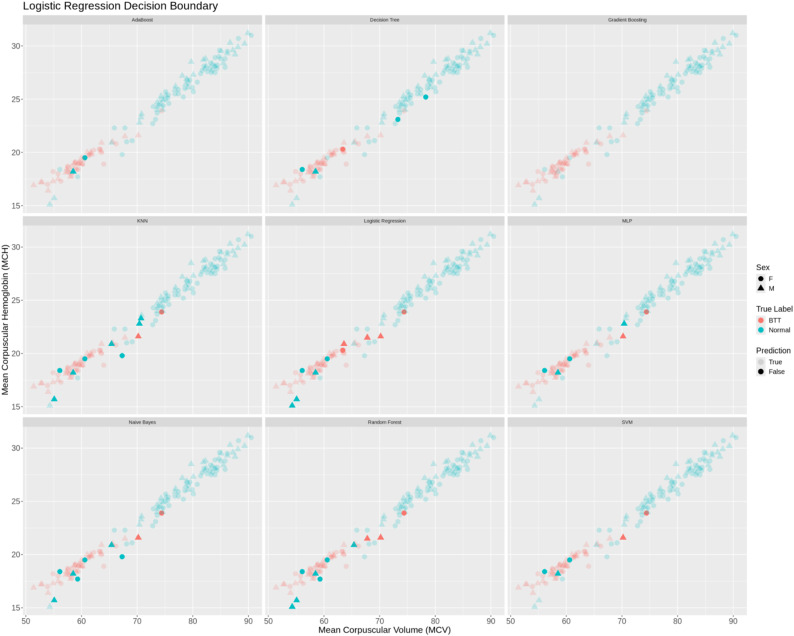
Classification of β-thalassemia trait (BTT) versus normal cases using only MCV and MCH as input features. The scatter plots illustrate the distribution of true positives (BTT) and true negatives (Normal) predicted by each model, demonstrating that MCV and MCH alone provide substantial discriminatory power for preliminary screening

To further illustrate this, all nine algorithms were initially trained using only MCV and MCH as input features. The resulting classification performance is shown in Fig. 4, which displays the separation of positive and negative cases by each algorithm in the test set. The graph demonstrates that even with just these two parameters, the models are able to distinguish BTT-positive from normal cases with reasonable accuracy, highlighting the predictive power of MCV and MCH in preliminary screening. This also informed subsequent model development, where additional parameters were incorporated to further improve classification metrics such as sensitivity, specificity, and overall accuracy.

Table 2 shows the performance metrics of various machine learning models trained on RBC parameters for differentiating BTT-positive (BTT) and normal cases. Overall, the models demonstrated high performance, with Classification Accuracies exceeding 92% across most configurations and AUC values consistently above 95%, reflecting strong discrimination between BTT-positive and normal cases. Sensitivity and specificity were well-balanced, with specificity ranging from approximately 93–94% and sensitivity from 88 to 94%, which is critical for a screening approach where minimising false negatives is essential. True positive counts (BTT → BTT) ranged from 48 to 50, and true negative counts (Normal → Normal) ranged from 84 to 85, indicating a low rate of misclassification. Models trained on slightly different subsets of RBC parameters, including age, sex, or history of iron supplementation, showed minor variations in metrics, suggesting robustness to feature selection. Among the algorithms, the MLP (100) model achieved the highest AUC (~ 96.88%) with well-balanced sensitivity and specificity, whereas Naive Bayes models exhibited the highest sensitivity (~ 92.45%) while maintaining competitive overall accuracy. Logistic Regression and SVM models also performed strongly and offer the additional advantage of interpretability.

**Table 2:**
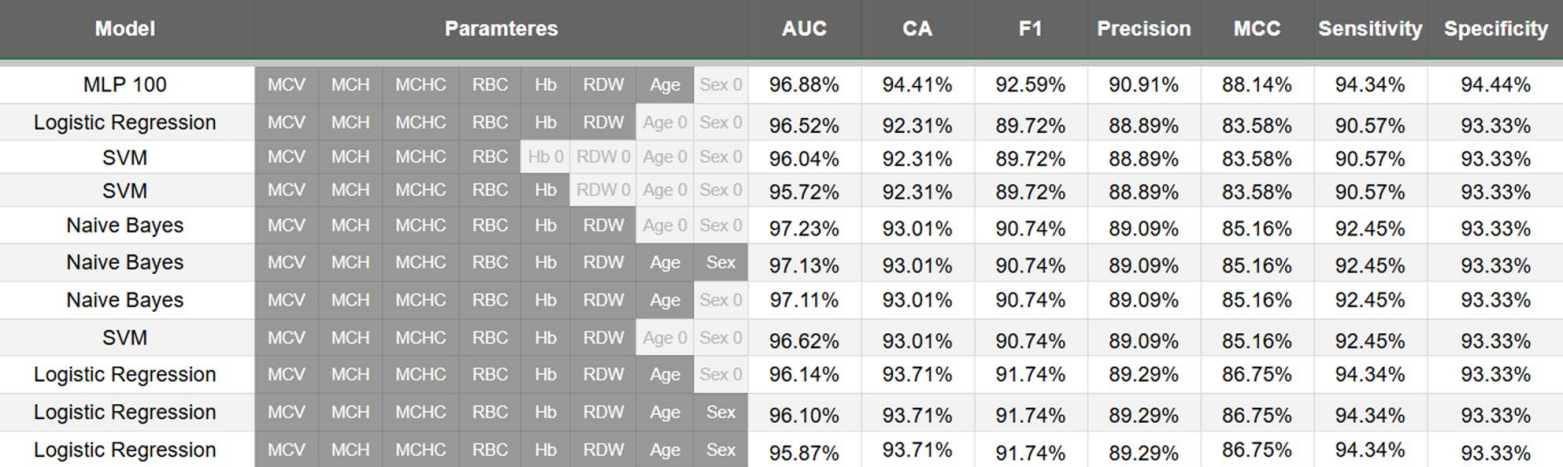
Performance metrics of various machine learning models trained on RBC parameters for differentiating BTT-positive (BTT) and normal cases

### Blood smear image analysis-only model

Table 3 summarises the performance of each CNN model on the independent test set, with all metrics reported along with their 95% confidence intervals.

**Table 3:** Performance of each CNN model on the independent test set, with all metrics reported along with their 95% confidence intervals

Model	CA (%)	F1 (%)	Precision (%)	Recall (%)	Specificity (%)	MCC (%)
MobileNetV2	37.2% (23.0–53.3)	52.6% (35.3–67.7)	35.7% (21.6–52.0)	100.0% (78.2–100.0)	3.6% (0.1–18.3)	0.113 (0.000–0.216)
ConvNeXtTiny	37.8% (23.8–53.5)	51.7% (34.6–65.7)	34.9% (21.0–50.9)	100.0% (78.2–100.0)	6.7% (0.8–22.1)	0.152 (0.000–0.264)
InceptionV3	60.5% (44.4–75.0)	63.8% (45.5–77.8)	46.9% (29.1–65.3)	100.0% (78.2–100.0)	39.3% (21.5–59.4)	0.429 (0.283–0.586)
VGG16	72.1% (56.3–84.7)	68.4% (48.5–83.3)	56.5% (34.5–76.8)	86.7% (59.5–98.3)	64.3% (44.1–81.4)	0.487 (0.229–0.715)
ResNet50	65.1% (49.1–79.0)	63.4% (43.9–79.1)	50.0% (29.9–70.1)	86.7% (59.5–98.3)	53.6% (33.9–72.5)	0.392 (0.131–0.624)
DenseNet121	62.8% (46.7–77.0)	61.9% (42.4–77.8)	48.1% (28.7–68.1)	86.7% (59.5–98.3)	50.0% (30.6–69.4)	0.362 (0.106–0.590)

VGG16 achieved the highest classification accuracy (72.1%) and the highest specificity (64.3%) among the models, while maintaining a strong recall (86.67%), indicating a balanced ability to identify both positive and negative samples. Models such as MobileNetV2, ConvNeXtTiny, and InceptionV3 reached perfect recall (100%), but their specificity was extremely low, leading to a high number of false positives and a risk of unnecessary follow-up testing. ResNet50 and DenseNet121 showed moderate performance across all metrics, offering more balanced but overall lower accuracy compared to VGG16.

McNemar’s test confirmed a statistically significant difference in classification performance between the Red Blood Cell Indices–based MLP and the VGG16 CNN image analysis model (*p* = 0.0078), driven primarily by the higher false-positive rate of the CNN.

Overall, the results demonstrate a clear trade-off between recall and specificity among the CNN models. Since the primary objective of this step is screening, avoiding missed positive cases is critical. Therefore, VGG16 was selected for blood smear analysis because it provides high sensitivity (recall 86.67%), ensuring that most potential β-thalassemia carriers are flagged for confirmation testing. Although this increases false positives, the pipeline tolerates this, as all flagged cases will undergo HPLC confirmation. This approach maximises patient safety and supports effective population screening within the dual-modal workflow.

Overall, the results demonstrate a clear trade-off between recall and specificity among the CNN models. Models such as ConvNeXtTiny and MobileNetV2 achieved very high recall, correctly identifying nearly all positive cases, but their extremely low specificity led to many false positives and unnecessary follow-up testing. In contrast, VGG16 provided the best balance, with the highest classification accuracy and specificity while maintaining high sensitivity (86.67%), reducing the risk of missed positive cases.

### Error analysis

To further interpret the performance of the proposed thalassemia screening pipeline, an error analysis was conducted to examine the characteristics and potential causes of model misclassifications. This analysis aimed to identify patterns among false positives and false negatives across both the RBC-based and image-based models, evaluate the complementary effects of the two-step approach, and reveal factors contributing to residual errors. Understanding these limitations provides valuable insight into model behaviour and guides future improvements in data preprocessing, feature selection, and threshold optimisation.

### Error analysis of red blood cell indices-based MLP model

**Fig. 5:**
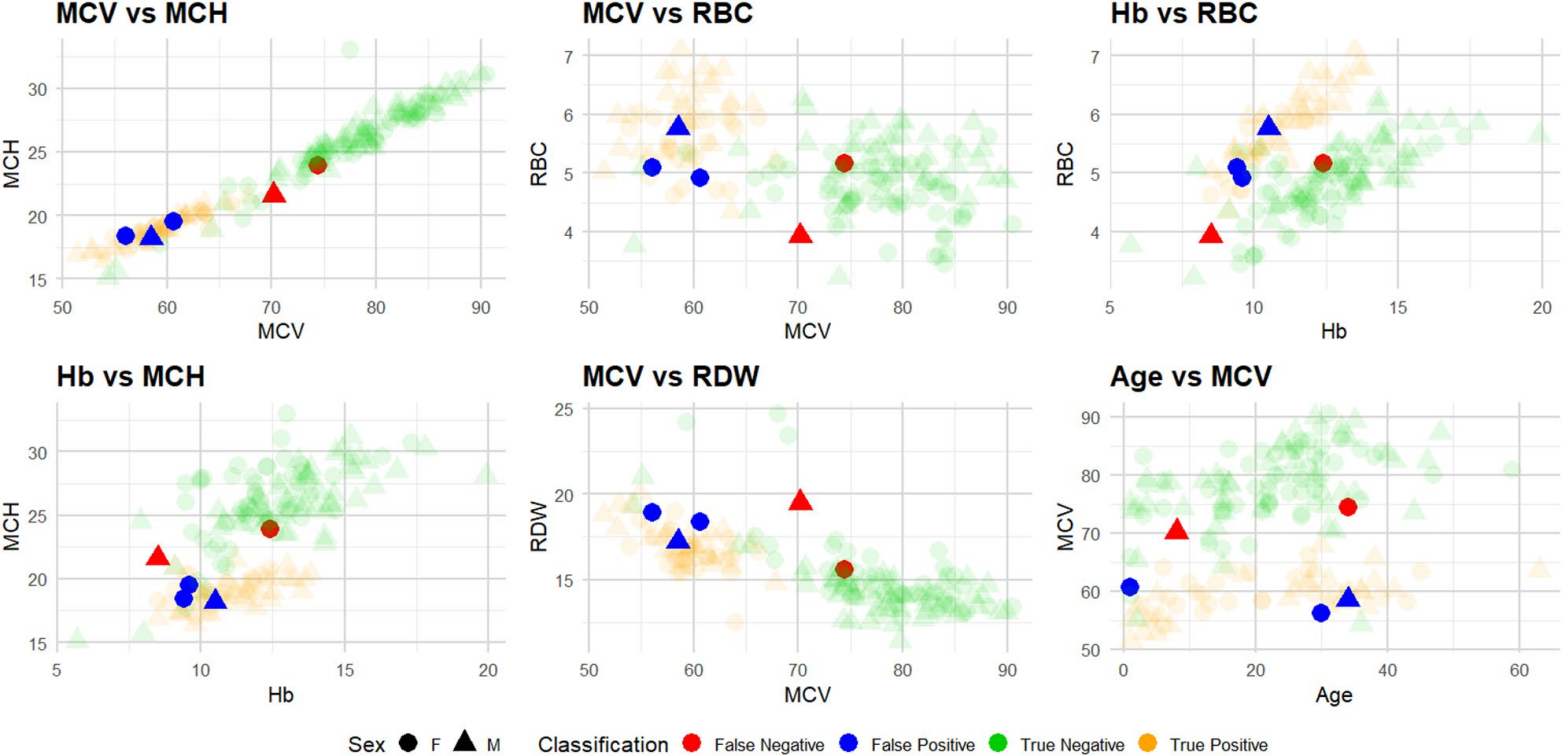
Multi-pair scatter plots showing MLP model predictions on the RBC dataset. True positives (orange), true negatives (green), false positives (blue), and false negatives (red) are displayed for each parameter pair (e.g., MCV vs. MCH, Hb vs. RBC). Point shapes indicate patient sex, and alpha values highlight misclassified samples

To further investigate the misclassifications of the Red Blood Cell Indices-based MLP model, scatter plots were generated for multiple pairs of haematological parameters. Each plot highlights true positives (TP), true negatives (TN), false positives (FP), and false negatives (FN), allowing visual assessment of the model’s decision boundaries and error patterns. The plots also indicate patient sex and adjust point transparency to emphasise misclassified cases. This multi-pair approach provides an intuitive way to explore the distribution of misclassified samples across different parameter combinations, identify regions where the model struggles, and guide potential improvements in feature selection or thresholding. Figure 5 shows Multi-pair scatter plots showing MLP model predictions on the RBC dataset. Visualisations illustrate the distribution of errors, reveal parameter regions contributing to misclassifications, and provide insights for refining the screening pipeline.

Analysis of the MLP misclassifications revealed that false negatives (FNs) cluster in regions of the MCV vs. MCH scatter plot that largely overlap with true negatives (TNs). These samples exhibited relatively higher MCV and MCH values compared to true positives, while their RBC counts were lower than those of typical positive cases but similar to those of negative samples. Biologically, this pattern may reflect borderline or atypical β-thalassemia carriers whose red blood cell indices do not fall within classical microcytic and hypochromic ranges. Such cases are known to occur in carriers with mild phenotypic expression, coexisting iron deficiency, or recent blood transfusions, leading to partial normalisation of haematological parameters. In the present dataset, the observed false negatives did not have recent blood transfusions or iron treatment, suggesting that their borderline haematological profiles may be due to mild genetic variants, heterozygosity with less severe mutation expression, or individual physiological compensation mechanisms that partially normalise RBC indices. Consequently, the model may classify these borderline carriers as negative, illustrating the inherent limitation of using Red Blood Cell indices alone for precise detection in all patient subpopulations.

Conversely, false positives (FPs) were primarily located within regions characteristic of true positives, exhibiting lower MCV and MCH values alongside higher RBC counts. This pattern suggests that while these individuals do not carry the β-thalassemia trait, their haematological parameters resemble those of carriers, possibly due to benign microcytosis, variations in haemoglobin concentration, or other haematological conditions. Such overlap underscores the trade-off between sensitivity and specificity in screening approaches and highlights the necessity of confirmatory testing to accurately distinguish true carriers from individuals with overlapping but non-pathological profiles.

### Error analysis of image-based model (VGG-16)

To further interpret the performance of the image-based screening step, misclassifications from the VGG-16 model were examined. False negatives (FNs) and false positives (FPs) were analysed in relation to morphological characteristics observed in the blood smear images, providing insight into patterns that contribute to residual errors. This analysis aimed to identify visual features associated with borderline cases, evaluate limitations in feature extraction by the CNN, and highlight scenarios where morphological ambiguity affects predictive accuracy.

**Fig. 6:**
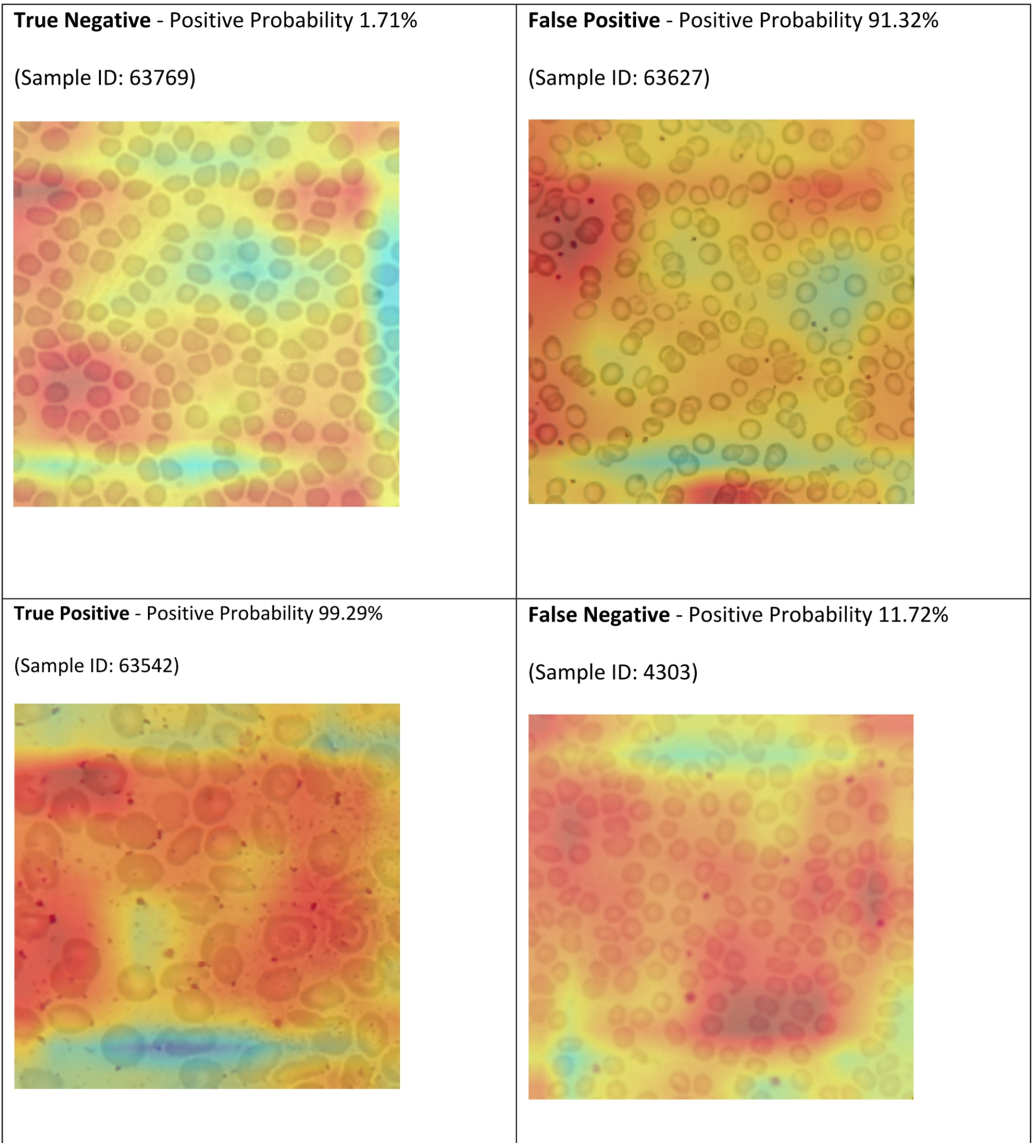
Grad-CAM visualisations were generated for representative true negative (TN), true positive (TP), false positive (FP), and false negative (FN) samples

Grad-CAM visualisations were generated for representative true negative (TN), true positive (TP), false positive (FP), and false negative (FN) samples to investigate the image-based model’s attention patterns (Fig. 6). For the TP sample (ID: 63542, Positive Probability: 99.29%), attention was strongly focused on the central regions of RBCs, particularly highlighting morphological features consistent with β-thalassemia. Similarly, the TN sample (ID: 63769, Positive Probability: 1.71%) showed minimal attention on RBCs, with the model correctly ignoring normal cell morphology. In contrast, the FP sample (ID: 63627, Positive Probability: 91.32%) exhibited concentrated attention on RBC clusters, likely misinterpreting normal variations or overlapping cells as thalassemia-like features, resulting in high predicted probability despite the sample being negative. The FN sample (ID: 4303, Positive Probability: 11.72%) displayed diffuse and less distinct attention across RBCs, indicating that subtle or borderline morphological abnormalities were insufficient for confident detection. Collectively, these patterns suggest that misclassifications arise from either over-interpretation of normal variations (FPs) or under-recognition of mild, atypical morphological features (FNs), highlighting inherent limitations of relying solely on image morphology for screening in borderline cases.

### Probabilistic calibration of MLP and CNN models

**Fig. 7:**
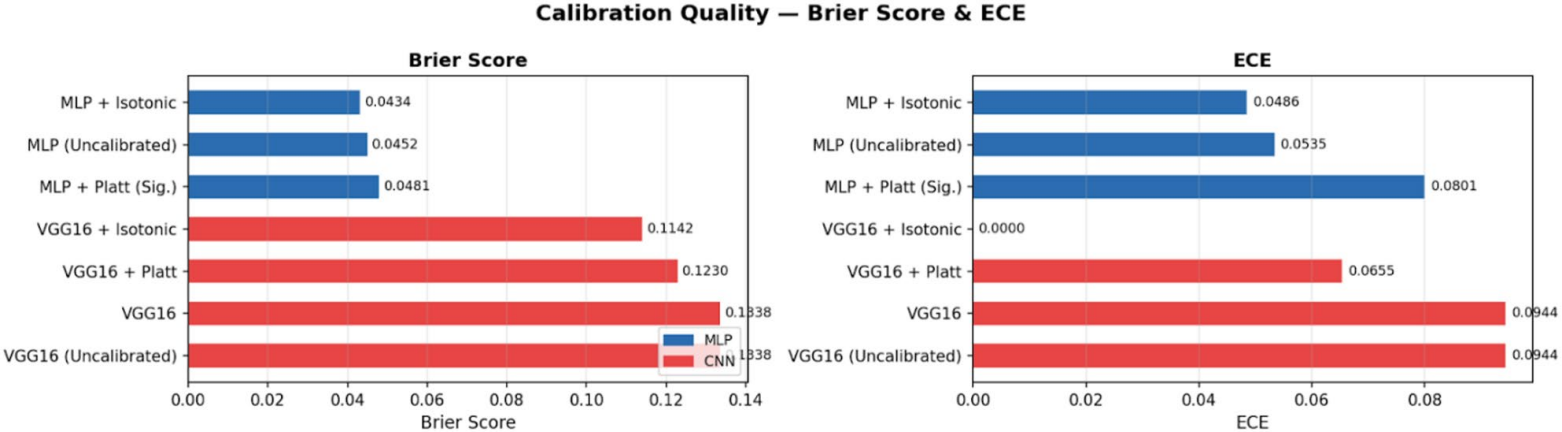
Calibration quality- brier score

As summarized in Table 1; Fig. 7, the MLP achieved lower Brier scores than the CNN, indicating higher overall probabilistic accuracy on this dataset. Calibration methods had a more substantial effect on CNN predictions than on the already well-calibrated MLP, demonstrating the importance of calibration for overconfident models.

**Table 4:** Probability Calibration Performance for MLP and VGG16 Models Using Brier Score and Expected Calibration Error

Model/Variant	Brier Score	ECE	Group
MLP + Isotonic	0.0434	0.0486	MLP — 10-Fold Cross-Validated Probabilities
MLP (Uncalibrated)	0.0452	0.0535	MLP — 10-Fold Cross-Validated Probabilities
MLP + Platt (Sig.)	0.0481	0.0801	MLP — 10-Fold Cross-Validated Probabilities
VGG16 + Isotonic	0.1142	0	CNN Models — Validation Set Probabilities
VGG16 + Platt	0.123	0.0655	CNN Models — Validation Set Probabilities
VGG16	0.1338	0.0944	CNN
VGG16 (Uncalibrated)	0.1338	0.0944	CNN Models — Validation Set Probabilities

The MLP model is inherently accurate and reasonably calibrated, with isotonic regression providing minor improvements. The VGG16 CNN, in contrast, initially produced poorly calibrated probabilities; isotonic regression substantially corrected this, producing probability estimates closely aligned with observed outcomes. These results support the use of isotonic calibration for overconfident CNN models while indicating that MLP predictions are already trustworthy.

### Evaluation of alternative sequential screening pipelines

To further evaluate the robustness and optimal configuration of the dual-modal screening system, two alternative pipeline architectures were tested by altering the order of Red Blood Cell Indices and image-based analysis. This served as an ablation-style assessment to determine how sequencing the analytical stages influences diagnostic accuracy, sensitivity, and specificity. Sensitivity was prioritised to minimise false negatives, while specificity, F1-score, and ROC-AUC provided complementary measures of classification balance and discriminative ability.

### Pipeline 1 (Red blood cell indices analysis → image analysis)

In the RBC → Image sequential pipeline, 97 patients were classified as negative in Step 1 and proceeded to image-based evaluation. Among these, 6 were true β-thalassemia carriers and 91 were true non-carriers. The VGG-16 model correctly identified all 6 carriers (sensitivity = 100%), although the confidence interval was wide due to the small number of positive cases in this subset. This reflects the intended design of the sequential screening workflow, where the majority of carriers are detected in the first stage.

In this configuration, the Multi-Layer Perceptron (MLP) model based on full blood count parameters was applied first. Samples classified as negative were subsequently analysed using the VGG-16 CNN model. This approach aimed to minimise the number of smear analyses required while ensuring that no β-thalassemia carriers were missed. The combined pipeline achieved 100% sensitivity and 60.7% specificity, indicating that all positive cases were successfully detected but at the cost of increased false positives. Importantly, this configuration reduced the overall need for smear preparation and image processing by 37.7%, representing a practical advantage in large-scale screening workflows.

Table 4 shows the performance metrics of pipeline 1. All metrics reported along with their 95% confidence intervals.

**Table 5:** Performance metrics of the Red Blood Cell Indices → Image Analysis pipeline for β-thalassemia screening. The MLP model was applied to Red Blood Cell parameters first, followed by VGG-16–based image analysis for Red Blood Cell indices analysis-negative cases

Step	Model	Sensitivity (%)	Specificity (%)	Classification Accuracy (%)
1	Red Blood Cell Indices Analysis (MLP) only	88.2% (63.6–98.5%)	92.9% (76.5–99.1%)	91.1% (78.8–97.5%)
2	Image Analysis (VGG-16) only	100.0% (15.8–100%)	57.7% (36.9–76.6%)	60.7% (40.6–78.5%)
Overall Pipeline		100% (80.5–100%)	60.7% (40.6–78.5%)	75.6% (60.5–87.1%)

**Fig. 8:**
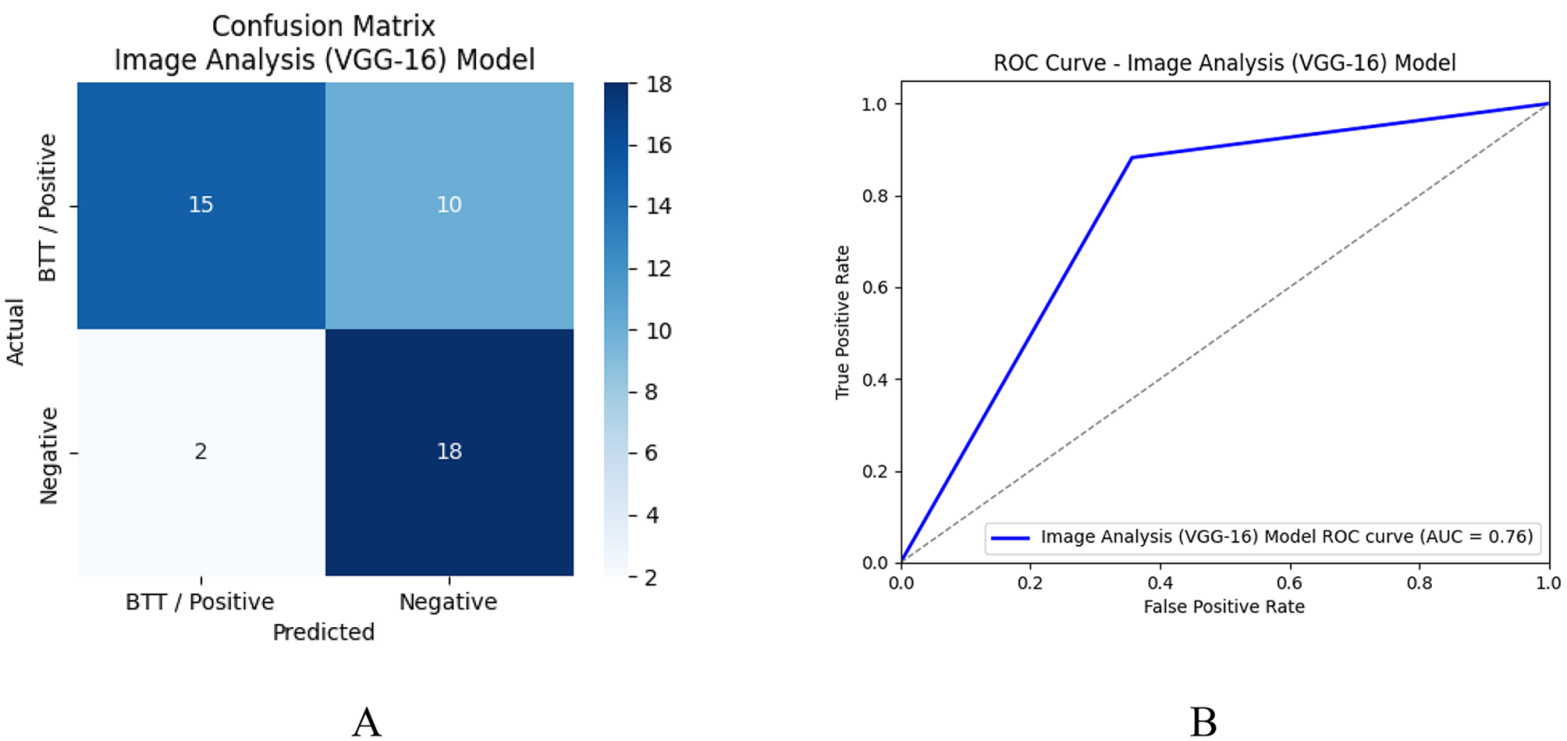
Confusion Matrix for Peripheral Blood Smear Analysis – VGG16 CNN Model (**A**) and ROC Curve for Peripheral Blood Smear Analysis – VGG16 CNN Model(**B**)

Figure 8 shows the Confusion matrices and the ROC curve for the image analysis model.

### Pipeline 2 (Image analysis → red blood cell indices analysis)

The second configuration reversed the order, applying VGG-16–based image analysis first and subsequently re-evaluating negative cases with the MLP model. This sequence also achieved 100% sensitivity, confirming that no positive samples were missed. Although specificity increased slightly to 64.3%, all samples required blood smear preparation and image processing. This introduced significant manual effort, time consumption, and computational workload. Consequently, despite maintaining perfect sensitivity, the operational overhead makes this architecture less practical for large-scale or resource-limited screening programs. Table 5 shows the performance metrics of the Image Analysis → Red Blood Cell Indices analysis pipeline for β-thalassemia screening.

**Table 6:** Performance metrics of the Image Analysis → Red Blood Cell Indices analysis pipeline for β-thalassemia screening. VGG-16–based image analysis was applied first, followed by MLP analysis for image-negative cases

Step	Model	Sensitivity (%)	Specificity (%)	Classification Accuracy (%)
First step	Image Analysis (VGG-16)	88.2% (63.6–98.5%)	64.3% (44.1–81.4%)	73.3% (58.1–85.4%)
Second step	Red Blood Cell Indices Analysis (MLP)	100.0% (15.8–100.0%)	94.4% (72.7–99.9%)	95% (75.1–99.9%)
Overall Pipeline		100.0% (80.5–100.0%)	64.3% (44.1–81.4)	77.8% (62.9–88.8%)

**Fig. 9:**
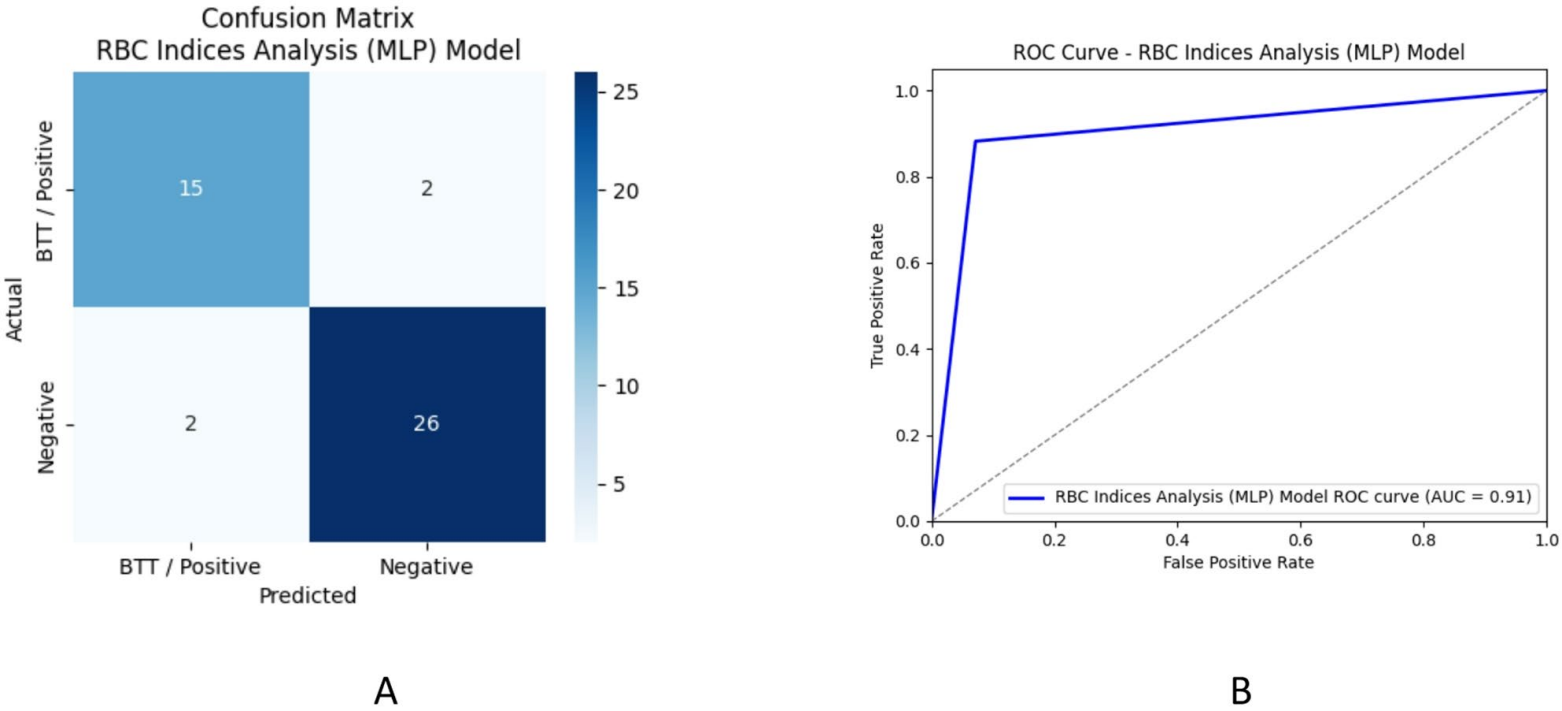
Confusion Matrix for Red Blood Cell Indices Analysis – MLP Model (**A**) and ROC Curve for Red Blood Cell Indices Analysis – MLP Model (**B**)

Figure 9 shows the Confusion matrices and the ROC curve for the MLP model.

### Comparative interpretation

Both pipelines maintained perfect sensitivity, confirming the value of combining haematological and morphological features for comprehensive β-thalassemia screening. However, starting with Red Blood Cell indices analysis followed by image evaluation of negatives provided greater operational efficiency by reducing smear preparation and processing requirements. This indicates that the Red Blood Cell Indices → Image pipeline is more suitable for scalable, resource-conscious screening in clinical and public health settings.

### Overall dual-modal pipeline performance

The proposed dual-modal screening pipeline combines Red Blood Cell indices analysis and blood smear image classification to maximise sensitivity while reducing unnecessary laboratory workload. In this study, all positive cases (BTT carriers) were successfully identified, with zero missed cases, demonstrating that the pipeline is highly reliable for screening purposes. By first using the Red Blood Cell indices analysis model to classify cases, only the negative cases from the first step were forwarded to blood smear analysis. Consequently, the number of blood smear preparations was reduced from 152 total samples to 97, effectively saving 37% of laboratory time, reagents, and personnel effort.

This two-step approach ensures that almost all positive cases are flagged for confirmation while minimising unnecessary laboratory procedures, making the pipeline both practical and cost-effective, particularly in settings with limited access to haematologists or specialised laboratory infrastructure. Additionally, by using a high-sensitivity VGG16 model for the second step, the pipeline preserves patient safety without compromising screening efficiency.

### Model explainability with SHAP

**Fig. 10:**
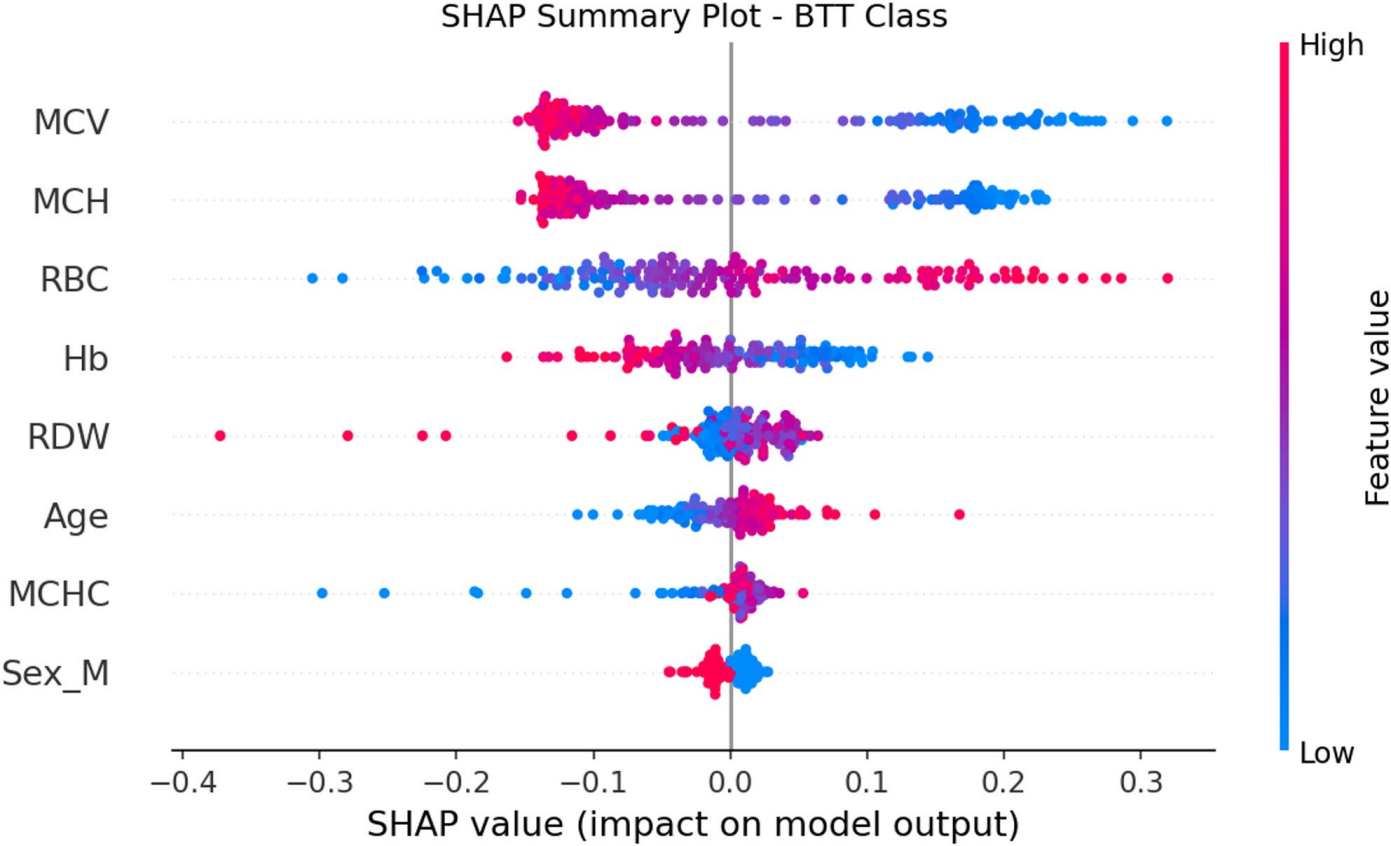
SHAP summary plot showing the direction and magnitude of each feature’s contribution to the MLP model’s β-thalassemia trait predictions

Figure 10 illustrates the SHAP summary plot for the MLP model predicting β-thalassemia trait. Features are ordered by overall importance, with MCV, MCH, RBC, and Hb emerging as the strongest contributors. Higher values of MCV and MCH (shown in blue) generally decrease the predicted probability of BTT, whereas lower values (pink/red) push the prediction toward the BTT class—consistent with microcytic, hypochromic patterns in β-thalassemia carriers. RBC shows the opposite trend: higher RBC counts increase the probability of BTT, reflecting the characteristic elevated RBC indices in carriers. RDW, age, MCHC, and sex show smaller but notable effects. Overall, the SHAP plot demonstrates that the model’s learned patterns align with known hematological signatures of β-thalassemia trait, providing interpretability and clinical plausibility.

## Discussion

This study demonstrates the feasibility and potential utility of a dual-modal machine learning pipeline for β-thalassemia screening in Sri Lanka, combining Red Blood Cell (RBC) indices and peripheral blood smear images. By integrating these two complementary modalities, the system leverages both quantitative haematological features and morphological patterns, providing a sensitive, cost-effective, and scalable approach suitable for deployment in low-resource settings.

Currently, MCV < 80 fL and MCH < 27 pg are used as cutoff values in screening programs, below which confirmatory investigations are recommended. However, iron deficiency anaemia is several-fold more prevalent than thalassemia trait, resulting in a substantial proportion of individuals with coexisting iron deficiency and thalassemia trait. In this context, commonly used discrimination indices such as the Mentzer index and Shine & Lal index, although useful as initial screening tools, demonstrate reduced diagnostic accuracy in the presence of iron deficiency. Peripheral blood film examination often provides more reliable morphological clues in such situations and supports the pragmatic strategy of administering a three-month therapeutic trial of iron followed by reassessment, particularly in resource-limited settings where serum ferritin estimation is not readily available. However, limitations in trained human resources render routine blood film examination impractical during large-scale screening of school children and community populations. Therefore, this newly developed tool is of particular importance, as it enables screening through a blood film–equivalent assessment, effectively addressing both the diagnostic limitations of existing indices and the operational challenges inherent in mass screening programs. It leverages a multidimensional set of RBC indices through an MLP model and complements this with CNN-based smear analysis.

While earlier studies have applied machine-learning methods to either RBC indices or smear images, this framework introduces a sequential fusion strategy that combines two independent models—a multi-feature MLP trained on RBC indices and a CNN trained on smartphone-captured smear images—to maximise sensitivity while reducing unnecessary smear preparation. It improved clinical performance by achieving 100% sensitivity in the combined pipeline while also enhancing operational efficiency by reducing smear preparation and review by 37.7%.

A sensitivity-oriented assessment of feature selection was performed by comparing model performance across multiple feature subsets. PCA and individual feature contribution analyses identified MCV and MCH as the strongest discriminators.

### Diagnostic performance and key findings

Analysis of the RBC dataset revealed clear microcytic and hypochromic patterns among β-thalassemia trait (BTT) carriers, characterised by lower MCV (59.89 ± 4.20 fL) and MCH (19.05 ± 1.37 pg) and elevated RBC counts (5.72 ± 0.65 × 10¹²/L), compared to non-carriers. RDW was also higher among carriers, reflecting increased variability in red cell size. These differences provided a strong discriminatory signal for the initial RBC-based screening step. The MLP machine learning classifier, optimised using these parameters, achieved high specificity, allowing positive cases to be confidently referred for confirmatory HPLC testing while minimising unnecessary smear preparation for individuals screened as negative.

The blood smear image analysis component further enhanced screening sensitivity, effectively detecting subtle morphological changes, including target cells, pencil cells, and other characteristic features of thalassemia. Using the VGG16 CNN architecture with transfer learning, the system reliably identified borderline and atypical carriers that could be missed by RBC indices alone. The integration of structured and image-based data in a sequential pipeline ensured that no true positive cases were overlooked, while maintaining operational efficiency by limiting smear analysis to individuals not flagged in the first step.

### Practical implications

This dual-modal approach addresses several challenges faced by national screening programmes in low- and middle-income countries. Firstly, it reduces reliance on specialised laboratory equipment, as smartphone-assisted microscopy provided sufficiently high-quality images. Secondly, the sequential workflow optimises resource utilisation by minimising the number of smears requiring manual examination, thereby saving time, reagents, and labour. Thirdly, given the limited number of haematologists in Sri Lankan hospitals, conducting mass screening is extremely tiring and exhausting for available staff; the model’s portability and compatibility with widely available smartphones enable decentralised screening in rural and primary care settings. This aligns with ongoing efforts to expand thalassaemia prevention programmes in Sri Lanka, including premarital screening initiatives.

By combining RBC indices and smear morphology, the system also improves diagnostic confidence, reducing false positives and negatives compared to single-modality approaches. This is particularly relevant in LMICs, where conventional confirmatory tests like HPLC or genetic analysis may be limited by cost, infrastructure, or accessibility. The proposed pipeline could serve as a triage tool, prioritising individuals for confirmatory testing while reducing the burden on central laboratories.

### Limitations

Despite its promising performance, the study has several limitations that should be considered when interpreting the results. The dataset was derived from a single tertiary referral centre in Kurunegala, limiting geographical and ethnic diversity. The independent test set was relatively modest in size. Consequently, the reported performance metrics should be interpreted as preliminary estimates of model performance. While age and sex distributions were balanced, other demographic factors, such as socioeconomic background, ethnicity, and regional prevalence variations, were not captured. Although the results demonstrate promising sensitivity for β-thalassemia carrier detection, further validation using larger, multi-centre datasets representing diverse populations and laboratory settings will be necessary to confirm the robustness and generalisability of the proposed screening pipeline. Such external validation will be essential before considering broader clinical deployment in national screening programmes.

Although smartphone-assisted image acquisition reflects real-world conditions, image quality may vary depending on operator experience, lighting, and device specifications. Data augmentation and CNN training mitigated some of these effects; however, further field validation is necessary to assess robustness under variable community-level conditions.

The current pipeline remains a research prototype and has not yet undergone regulatory review, integration with laboratory information systems, or prospective clinical evaluation.

### Future directions

Future work should focus on expanding the dataset to include multi-centre, nationwide samples and additional demographic variables, enhancing model generalisability and robustness. Prospective studies are also required to evaluate real-world usability, workflow integration, and the impact on screening coverage, particularly in the context of mass thalassaemia prevention programs. Also multi-centre, external validation to assess robustness across diverse demographic and operational environments is recommended.

Engagement with policymakers, healthcare administrators, and frontline health workers will be essential to understand operational constraints, gather feedback, and secure support for implementation. The development of a clinician-friendly mobile application with streamlined reporting could facilitate adoption, improve operational efficiency, and enable decentralised screening in rural and primary care settings.

The dual-modal framework could be adapted for other haemoglobinopathies, supporting broader public health screening initiatives and contributing to scalable, sustainable, and equitable approaches to genetic disorder prevention.

### Implementation and ethical considerations

While the dual-modal machine learning pipeline demonstrates high sensitivity and practical feasibility, several considerations must be addressed prior to deployment.

#### Data security and privacy

The system relies on patient data and blood smear images, which are sensitive health information. Secure storage, anonymisation, and controlled access are essential to prevent unauthorised use or breaches. Any mobile or cloud-based deployment must comply with national and international data protection regulations, including secure data transmission and storage protocols.

#### Operator and workflow variability

The quality of smartphone-assisted images may vary with operator skill, lighting, and device type. Standardised training, clear imaging protocols, and continuous quality control are necessary to maintain model reliability across diverse settings.

#### Ethical and regulatory oversight

Clinical deployment must align with ethical guidelines, obtain regulatory approvals, and integrate seamlessly with existing laboratory and screening workflows. Continuous monitoring for potential biases, misclassifications, or inequitable outcomes is essential, particularly when expanding to new populations.

#### Stakeholder engagement

Successful implementation requires input from healthcare workers, administrators, and policymakers to ensure the system is feasible, acceptable, and sustainable within national screening programmes. Addressing practical concerns, such as workload, cost, and training, will be critical for uptake and long-term adoption.

#### Multi-modal learning framework

Future work will focus on developing an end-to-end multi-modal fusion framework that jointly integrates red cell indices and smartphone microscopy images to enhance diagnostic performance. Advances in multi-modal medical image fusion demonstrate that deep, feature-level and decision-level fusion can capture complementary diagnostic information and improve model robustness *(Zubair et al.*,* 2025)*. Incorporating such fusion strategies is expected to further reduce false negatives and improve generalizability across diverse β-thalassemia cohorts.

#### Self-supervised contrastive pretraining

We also plan to incorporate self-supervised contrastive pretraining techniques to improve feature robustness in the image encoder, as such methods (e.g., SimCLR- or MoCo-based frameworks) have shown strong potential for reducing variance and enhancing generalization in settings with limited labeled medical images.

#### Synthetic image augmentation

To mitigate overfitting associated with the limited sample size, future extensions will explore synthetic image augmentation using generative models (e.g., GAN-based synthesis) and transfer learning from larger publicly available hematology and microscopy datasets, which may enhance feature robustness and improve generalization across sites.

Proactively addressing these considerations will enable safe, effective integration of the system into national screening pathways while maintaining patient trust and clinical reliability.

### Conclusion

This study demonstrates that a dual-modal machine learning pipeline combining RBC indices and peripheral smear morphology can provide a sensitive, potential to be cost-effective, and practical approach for β-thalassemia screening. By enabling early detection and prioritisation of at-risk individuals, the system has potential to strengthen national prevention programs, reduce healthcare burden, and support decentralised screening in low-resource settings. It’s design, optimised for smartphones and minimal infrastructure, illustrates a scalable model for implementing AI-driven diagnostic support in LMICs.

## Supplementary Information

Below is the link to the electronic supplementary material.

Supplementary Material 1

## Data Availability

The datasets used during the current study are available from the corresponding author on reasonable request.
