# Supplementary material for: A dual-modal machine learning framework integrating red blood indices and smartphone-captured microscopic images for β-thalassemia screening
Source: BMC Med Inform Decis Mak. 2026 Mar 21;26:148. doi: 10.1186/s12911-026-03451-y (PMC13130607; doi:10.1186/s12911-026-03451-y)
Supplement: Supplementary file 1 — Supplementary Material 1 [file 12911_2026_3451_MOESM1_ESM.pdf]

Reproducibility package

Input Features

The RBC-based model utilised eight haematological and demographic parameters:

- Mean corpuscular volume (MCV)
- Mean corpuscular haemoglobin (MCH)
- Mean corpuscular haemoglobin concentration (MCHC)
- Red blood cell count (RBC)
- Haemoglobin concentration (Hb)
- Red cell distribution width (RDW)
- Age
- Sex (Female = 0, Male = 1)

Missing Data Handling

Samples with incomplete full blood count measurements or untraceable confirmatory HPLC diagnoses were excluded prior to analysis. No post-hoc imputation was performed.

Data Scaling

Continuous numerical features were standardised using **StandardScaler**, fitted exclusively on the training dataset. Identical transformation parameters were subsequently applied to validation and independent test datasets to prevent information leakage.

Classification Threshold

Carrier status was determined using a fixed probability threshold of **0.5** for all reported performance metrics.

Hyperparameter Tuning

Hyperparameter Optimisation

Hyperparameters for each algorithm were tuned using grid search or random search to achieve optimal predictive performance. Each architecture was fine-tuned by replacing the final fully connected layers with custom classification layers tailored to the binary task (carrier vs. non-carrier). Key hyperparameters such as learning rate, batch size, number of trainable layers, optimizer choice, and dropout rate were systematically varied to identify the optimal configuration for each model.

MLP Model (haematological data):

- Input features: MCV, MCH, RBC, RDW, Hb, MCHC, Age, Sex

- Hidden layers and neurons: Optimised iteratively via grid search (final: 1 hidden layer with 100 neurons)
- Activation function: ReLU (activation='relu')
- Solver/optimizer: Adam (solver='adam')
- Learning rate: adaptive, initial = 0.001
  Maximum iterations: 200 (max_iter=200)
- L2 regularisation: alpha=0.0001
- Batch size: 32, chosen based on training set size
- Stratified 10-fold cross-validation (StratifiedKFold(n_splits=10, shuffle=True, random_state=42)) to ensure balanced representation of carriers and non-carriers and prevent data leakage

CNN Model (blood smear images, VGG16-based):

- Base model: VGG16 with ImageNet weights; **all convolutional layers frozen**, custom head trainable for binary classification
- Input size: 224 × 224 × 3
- Optimizer: Adam (learning_rate=0.001)
- Batch size: 16
- Epochs: 100
- Early stopping: patience = 5 epochs, based on validation loss
- Patient-level evaluation: per-patient predictions obtained by averaging all image predictions; classification threshold = 0.5
- Mixed precision training enabled (float16) for improved training efficiency

Reproducibility and Evaluation:

- Random seeds were fixed for NumPy and scikit-learn (random_state=42) to ensure consistent results across runs.
- Predictions and probabilities were aggregated across folds, and evaluation metrics including sensitivity, specificity, accuracy, F1-score, ROC-AUC, and Matthews correlation coefficient were computed.
- All analyses and model training were conducted in Python. Outputs, including fold-level metrics, predictions, and probabilities, were saved for integration into the dual-modal pipeline.
- Performance visualisations across models were generated using Matplotlib and Seaborn to facilitate hyperparameter selection and model validation.
